# Novelty modulates human striatal activation and prefrontal–striatal effective connectivity during working memory encoding

**DOI:** 10.1007/s00429-018-1679-0

**Published:** 2018-05-11

**Authors:** Lena S. Geiger, Carolin Moessnang, Axel Schäfer, Zhenxiang Zang, Maria Zangl, Hengyi Cao, Tamar R. van Raalten, Andreas Meyer-Lindenberg, Heike Tost

**Affiliations:** 1 Department of Psychiatry and Psychotherapy, Central Institute of Mental Health, Medical Faculty Mannheim, University of Heidelberg, Mannheim, Germany; 2 Department of Psychiatry, Rudolf Magnus Brain Center, University Medical Center Utrecht, Utrecht, The Netherlands

**Keywords:** Corticostriatal circuits, Novelty, Dynamic causal modeling, fMRI

## Abstract

The functional role of the basal ganglia (BG) in the gating of suitable motor responses to the cortex is well established. Growing evidence supports an analogous role of the BG during working memory encoding, a task phase in which the “input-gating” of relevant materials (or filtering of irrelevant information) is an important mechanism supporting cognitive capacity and the updating of working memory buffers. One important aspect of stimulus relevance is the novelty of working memory items, a quality that is understudied with respect to its effects on corticostriatal function and connectivity. To this end, we used functional magnetic resonance imaging (fMRI) in 74 healthy volunteers performing an established Sternberg working memory task with different task phases (encoding vs. retrieval) and degrees of stimulus familiarity (novel vs. previously trained). Activation analyses demonstrated a highly significant engagement of the anterior striatum, in particular during the encoding of novel working memory items. Dynamic causal modeling (DCM) of corticostriatal circuit connectivity identified a selective positive modulatory influence of novelty encoding on the connection from the dorsolateral prefrontal cortex (DLPFC) to the anterior striatum. These data extend prior research by further underscoring the relevance of the BG for human cognitive function and provide a mechanistic account of the DLPFC as a plausible top-down regulatory element of striatal function that may facilitate the “input-gating” of novel working memory materials.

**Electronic supplementary material:**

The online version of this article (10.1007/s00429-018-1679-0) contains supplementary material, which is available to authorized users.

## Introduction

Corticostriatal circuits play an important role in the experience-dependent reorganization of human behavior and have been implicated in the formation of motor and cognitive symptoms in neuropsychiatric disorders including schizophrenia and Parkinson’s disease (Shepherd 2013). The cellular anatomy of the circuits consists of parallel feedback loops which interconnect the cortex, basal ganglia, thalamus, and frontal cortex in a topographically organized, functionally segregated, and integrative fashion (Alexander et al. 1986; Draganski et al. 2008) with spatially definable cognitive territories including the DLPFC at the cortical level and the dorsal anterior putamen and caudate head at the level of the striatum (Choi et al. 2012; Draganski et al. 2008; Postuma and Dagher 2006). A important node of this circuit is the striatum, which receives excitatory input from the cortex and connects to two basal ganglia pathways with opposite net effects on the downstream target sites in the thalamus and frontal cortex (direct-excitatory vs. indirect-inhibitory pathway). This connectivity pattern makes the striatum ideally suited to function as a filter, for example, by gating adequate movement programs to the motor cortex while preventing the execution of less appropriate and competing motor plans (Chevalier and Deniau 1990; Mink 1996).

Corticostriatal research has traditionally focused on movement control, action selection and the formation of procedural skills (Shepherd 2013). However, growing evidence supports an analogous role of the circuitry in cognitive function, in particular working memory. One line of evidence comes from clinical fMRI studies linking abnormal striatal neurotransmission, activation and connectivity to working memory deficits in disorders such as schizophrenia (Fusar-Poli et al. 2010; Quide et al. 2013; Simpson et al. 2010). Studies in healthy controls further highlight the relevance of basal ganglia activation for working memory encoding (Chang et al. 2007; McNab and Klingberg 2008; Moore et al. 2013), a task phase in which the “input-gating” of relevant materials (or filtering of irrelevant information) is an important mechanism to enhance working memory capacity (McNab and Klingberg 2008). Computational models assume a division of labor in the corticostriatal cognitive loop, with the DLPFC aiding robust maintenance of working memory content while the cognitive territories of the striatum (i.e., the dorsal anterior putamen and caudate head) support the dynamic updating of working memory buffers through the selective gating of task-relevant information (Frank et al. 2001; O’Reilly and Frank 2006; Schroll and Hamker 2013). Other data suggest that the striatal gating mechanism during working memory encoding may extend to other task-relevant stimulus attributes including the novelty or increased cognitive demands of the materials (Chang et al. 2007; Landau et al. 2004; Nee and Brown 2013). However, little is known about the relative contributions of the striatum to working memory function in the encoding relative to the retrieval phase of the task and for different degrees of stimulus novelty. Moreover, the proposed corticostriatal control mechanism that supports the proposed input-gating of relevant information during working memory encoding is still underexplored.

In this study, we aimed to elucidate the questions (1) of whether striatal activation during verbal working memory varies across task phase and novelty of the presented materials, and (2) by which corticostriatal connectivity mechanism the task-phase-specific engagement of the striatum during novelty processing is plausibly achieved. We used functional magnetic resonance imaging (fMRI) activation analyses and dynamic causal modeling (DCM) in 74 healthy volunteers performing a Sternberg working memory task with different task phases and degrees of stimulus familiarity. Based on the prior literature we expected more pronounced striatal activations in the encoding (vs. retrieval) phase of the task and during the processing of the cognitively more demanding novel (vs. practiced) Sternberg items. For the potential corticostriatal gating mechanism during the processing of novel working memory materials, we further aimed to explore whether the observed effects are indeed best explained by a model assuming a modulatory influence of stimulus encoding on the information flow between the DLPFC and the cognitive territories of the striatum, and if so, whether the winning model hypothesizes a top-down, bottom-up, or reciprocal increase in effective connectivity.

## Methods

### Participants

Seventy-four healthy right-handed subjects (43 females, age: 26 ± 7.0 years) participated in this study. Informed consent was obtained from all individual participants included in the study. The protocol was approved by the Ethics Committee of the Medical Faculty Mannheim at the University of Heidelberg. Subjects had no history of neurological illness, psychiatric disorders or substance abuse. Handedness was assessed using the Edinburgh Handedness Inventory (Oldfield 1971).

### fMRI experiment

**Fig. 1:**
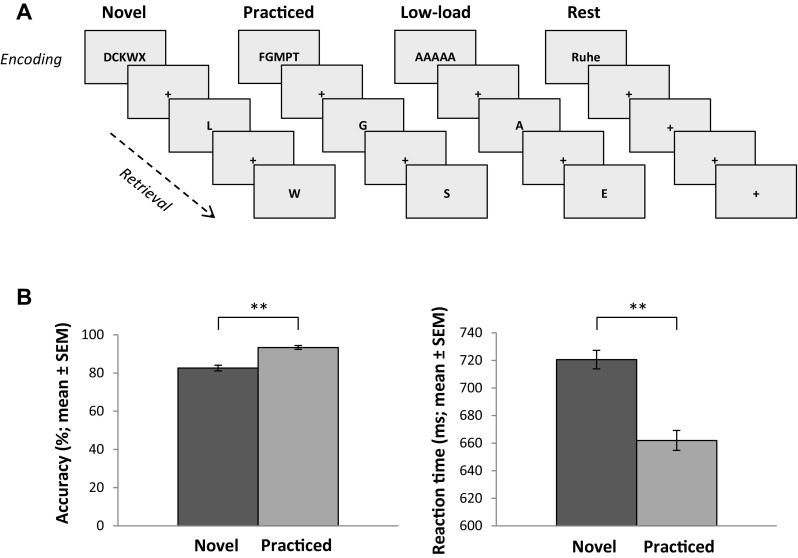
**a** Structure of the Sternberg task, which consisted of four different task conditions (novel, practiced, low-load cognitive control, and rest). **b** Behavioral results of the Sternberg task, with accuracy given in percent correct and reaction time given in milliseconds for the novel and practiced task conditions, respectively (**p < 0.001)

Subjects performed a modified Sternberg item recognition task with four task conditions and a total duration of 8.4 min (Fig. 1a). Similar paradigms have been previously used to study brain activations related to the processing of novel and automated stimulus–response relationships during verbal working memory (Jansma et al. 2001; van Raalten et al. 2008a, b). Each trial consisted of an encoding phase (3500 ms) during which a target set of five consonants was presented and had to be memorized. After a variable interstimulus interval (358–1790 ms, randomly jittered in steps of 358 ms), ten single-letter probes were presented consecutively for 1400 ms each, followed by a fixation cross for 800 ms in the retrieval phase (total duration of 26 s). For every single probe, the subjects had to indicate whether the letter was part of the target set (or not) by pressing the left (or right) button on an MR-compatible response pad.

Since our task aimed at investigating memory phase-dependent differences in the neural processing of novel and practiced working memory items, specific stimuli were trained and automatized prior to the fMRI scan. For this, subjects practiced three series of trials consisting of a fixed encoding stimulus set (i.e., FGMPT) and a fixed set of 50 letter probes (i.e., 25 target and 25 non-target probes) that were presented in a pseudo-randomized order in the retrieval phase. During the actual fMRI scan, novel and previously practiced working memory stimuli were presented along with a low-load cognitive control condition and a rest condition. While entirely novel target sets and probes were presented in the novel condition (e.g., DCKWX), the previously practiced target set and practiced probes were shown in the practiced condition. In the low-load cognitive control condition, the target set consisted of five identical vowels (AAAAA), and only two probes (one target and one non-target probe) were presented five times each during retrieval. In the rest condition, the German word for “rest” (Ruhe) was displayed instead of a target set followed by a fixation cross, and no response was required. All conditions were presented four times in a pseudo-randomized and counterbalanced order. To minimize condition-dependent differences in spatial attention and eye movements, all stimuli were presented at fixed positions on the screen. The basic motor and sensory processing demands of the novel and practiced conditions were comparable. Since our analyses were restricted to the direct comparison of novel and practice conditions, the low-load cognitive control condition and the rest condition were not analyzed further in this study.

### Behavioral data analysis

Mean reaction times (RT) for target and non-target stimuli were calculated for the novel and practiced conditions. Accuracy was recorded for each condition as percentage of correctly identified probes. We compared the performance parameters of the novel and practiced task conditions using paired sample *t* tests and applied a significance level of *p* < 0.05.

### Image acquisition

Functional data were acquired on a 3-T whole body MR Scanner (Siemens, Erlangen, Germany), with a 32-channel head coil [parallel imaging; generalized autocalibrating partially parallel acquisition (GRAPPA); iPAT = 2]. Functional images were acquired in a descending order with a gradient-echo echo-planar imaging (EPI) sequence (TR = 1790 ms, TE = 28 ms, flip angle = 76°, 34 axial slices, 3 mm slice thickness, 1 mm gap, matrix size: 64 × 64, field of view (FoV): 192 × 192 mm; whole brain coverage was ensured by tilting the FoV to − 25° from the individual anterior commissure–posterior commissure line).

### Image preprocessing

Data preprocessing was performed using standard routines of the Statistical Parametric Mapping software (SPM8; http://www.fil.ion.ucl.ac.uk/spm/software/spm8/). Briefly, this included a two pass realignment procedure (i.e., functional images were registered to the mean of the images after a first realignment to the first image), slice time correction, normalization to the Montreal Neurological Institute (MNI) EPI template (using the mean functional image as the source image and the MNI EPI template as the template image), and spatial smoothing with an 8-mm full-width at half-maximum (FWHM) Gaussian kernel. We additionally assessed mean framewise displacement to rule out excessive frame-to-frame motion in our sample. For this, we computed mean relative RMS (root mean squared) displacement according to Jenkinson et al. (2002). The resulting values (mean = 0.07 mm, SD = 0.03 mm, min = 0.03 mm, max = 0.21 mm) suggest that overall motion in our sample was low [see also Ciric et al. (2017)].

### Activation analysis

The activation analysis followed a two-level procedure in SPM8. At the first level, general linear models (GLM) were defined for each subject that included eight separate regressors for each stimulus type (novel, practiced, low-load cognitive control, and rest), and memory phase (i.e., encoding, retrieval). Regressors were modeled using delta (stick) functions for the encoding phases and boxcar functions for the retrieval phases. To account for head motion, the six head motion parameters from the realignment step were included as nuisance covariates into the model. During model estimation, the data were high-pass filtered with a cutoff of 128 s and an autoregressive model of the first order was applied. Contrast images were calculated for each subject to assess the (1) main effect of memory phase (encoding > retrieval, retrieval > encoding); (2) main effect of stimulus type (novel > practiced, practiced > novel), and (3) the encoding specific effect of stimulus type (encoding-novel > encoding-practiced, encoding-practice > encoding-novel). The first-level contrast images were entered into second-level random-effects models using age and sex as covariates of no interest, and one-sample *t * tests were calculated for statistical inference at the group level (*p* < 0.05, whole brain family-wise error (FWE) corrected). Note that results for the main effect of memory phase are reported in the supplemental material (Table S1, Figure S1) since the direct comparison of encoding and retrieval phases is of limited interpretability given profound differences in visual stimulation, task demands, and duration.

### Dynamic causal modeling

In addition to the activation analysis, we used DCM to explore the proposed role of prefrontal–striatal interactions for the regulation of the access of novel information into working memory. In brief, DCM allows clarifying how a specific brain region intrinsically exerts influences on another brain region and how this influence is modulated by the experimental conditions of a task. DCM models three brain dynamics in the context of external stimuli (Friston et al. 2003; Stephan et al. 2007): the endogenous coupling between two regions (intrinsic connections), the impact of experimental conditions on the regions themselves (driving inputs) and on the strength of the coupling between the regions (modulatory effects).

Based on the published literature (Gruber et al. 2006; O’Reilly and Frank 2006) and our own activation findings, we focused on the functional interaction of two key cognitive nodes in the cortical-striatal circuitry, the DLPFC and the downstream input node for excitatory projections from the prefrontal cortex at the level of the basal ganglia in the anterior striatum. Specifically, we aimed at examining potential modulatory effects of the encoding phase of novel and practiced items on the DLPFC and its effective connectivity to the striatum in the context of this Sternberg working memory task.

#### Definition of subject-specific volumes of interest (VOIs)

We defined subject-specific VOIs in two steps. First, we derived two anatomical masks from the Automated Anatomical Labeling (AAL) Atlas (Tzourio-Mazoyer et al. 2002) representing the DLPFC and the striatum. The DLPFC mask covered the AAL regions of Brodmann area (BA) 46, whereas the striatum mask covered the merged AAL regions of the putamen and caudate nucleus. Since we focused on the dorsolateral prefrontal loop (executive loop) within the corticostriatal circuits (Alexander et al. 1986), only the anterior part of the putamen and the head of the caudate nucleus were chosen (MNI y ≥ −1) We subsequently defined subject-specific VOIs by superimposing the masks to the first-level statistical images of the “encoding-novel > encoding practice” contrast, identifying the peak statistical voxel within each mask, centering 6 mm spheres around the peak voxels, and extracting the first eigenvariate from these spheres. The VOI time series were adjusted for the effects of interest (EOI), which accounts for movement artifacts based on the realignment parameters and mean-corrects the data. Following previous literature demonstrating predominantly left hemispheric lateralization for verbal WM items (Nagel et al. 2013) and confirming a higher involvement of the left hemisphere during similar Sternberg tasks (Cairo et al. 2004; Chang et al. 2007; van Raalten et al. 2008a), we defined our VOIs within the left hemisphere. For an illustration of the distribution of individual VOIs, see Figure S2.

#### DCM model space definition and estimation

**Fig. 2:**
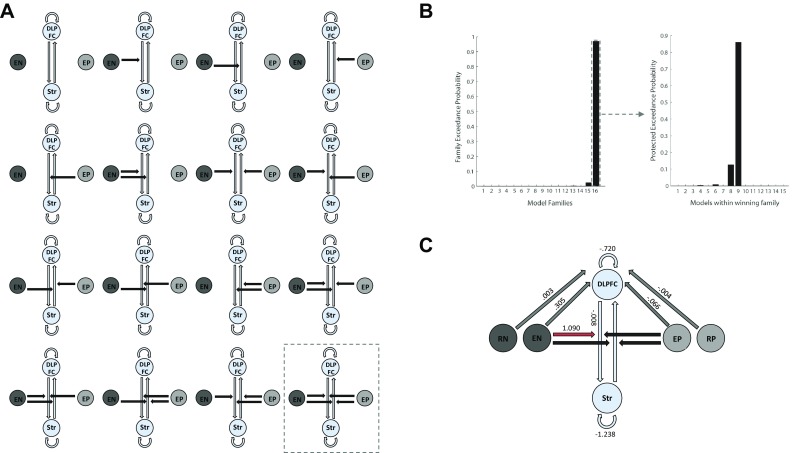
**a** Illustration of model families: bilateral intrinsic connections (light grey arrows) between DLPFC and striatum were fixed (i.e., not varied) across models. The two encoding conditions [encoding-novel (EN), encoding-practice (EP)] were defined as modulatory effects (black arrows), either jointly or separately, on the bidirectional intrinsic connections (DLPFC to striatum vs. striatum to DLPFC). Model families resulted from the variation of modulatory effects, individual models within each model family resulted from the variation of driving inputs (not shown). **b** Left: Bayesian model estimation (BMS) results for the model families. The winning model family (# 16: full modulation, i.e., modulation of both intrinsic connections by the two encoding conditions) was identified with a family exceedance probability of *p* = 0.94. Right: Bayesian parameter averaging (BPA) results within the winning family. The winning model (depicted in **c**) was identified with a protected exceedance probability of *p* = 0.86. **c** Illustration of the winning model. Driving inputs from all four conditions were directed to the DLPFC only. Parameter averages are only shown in case of statistical significance. The modulation of the connection from DLPFC to striatum is highlighted in red since this finding is central to our research question. For more information see Table 3. *EN* encoding novel, *EP* encoding practice, *RN* retrieval novel, *RP* retrieval practiced, *DLPFC* dorsolateral prefrontal cortex, *Str* striatum

We used the DCM12 toolbox implemented in SPM12 (r6685) for model definition and estimation of deterministic DCMs. In order to test whether the experimentally induced influence is consistent with a “top-down” and/or “bottom-up” regulatory effect, the following brain dynamics were included in the definition of DCM models. (1) Intrinsic connections: consistent with the anatomy of corticostriatal circuits (Alexander et al. 1986; Shepherd 2013) we assumed bidirectional intrinsic connections between the DLPFC and the basal ganglia. (2) Driving inputs: all four conditions (encoding-novel [EN], encoding-practice [EP], retrieval-novel [RN], retrieval-practice [RP]) were used as driving input to DLPFC, striatum or both (i.e., 2^4^ − 1 = 15; no input at all was ignored). (3) Modulatory effects: the two encoding conditions (EN, EP) were included as modulatory effects, either jointly or separately, on the bidirectional intrinsic connections (i.e., DLPFC to striatum vs. striatum to DLPFC; 2^4^ = 16). Systematic variation of these dynamics resulted in a total of 2^4^ * (2^4^ − 1) = 240 models. An illustration of all brain dynamics taken into account for model definition is provided in Fig. 2a.

In a first step, model families were defined by modulation patterns, resulting in 2^4^ = 16 families and compared using random effects Bayesian model selection (BMS) to identify the winning family. In a second step, the individual models within the winning family (15 models) were compared by means of random effects Bayesian model selection (BMS) to determine the model that most likely generated the observed data (winning model) assessed by the protected exceedance probability. The protected exceedance probability measures how likely any given model is more frequent than all other models in the comparison set and is, other than the exceedance probability per se, protected against the possibility that the alternative hypothesis is not true (Penny et al. 2010; Rigoux et al. 2014; Stephan et al. 2009, 2010).

In a last step, Bayesian parameter averaging (BPA) was used for a detailed description of the winning model.

## Results

### Behavioral data

During memory retrieval, subjects achieved accuracies of 82% [standard error of the mean (SEM) = 1.0%] in the novel condition and of 93% (SEM = 1.5%) in the practiced condition. Mean reaction times were 721 ms (SEM = 7 ms) for the novel condition and 662 ms (SEM = 7 ms) for the practiced condition (Fig. 1b). The statistical comparison confirmed a significant increase in accuracy (*t*_[73]_ = 8.9, *p* < 0.001) and a decrease in reaction time (*t*_[73]_ = 14.3, *p* < 0.001) during the retrieval of practiced stimuli relative to the retrieval of novel stimuli, consistent with a successful automatization of the trained working memory items prior to the scan.

### Activation analysis

**Fig. 3:**
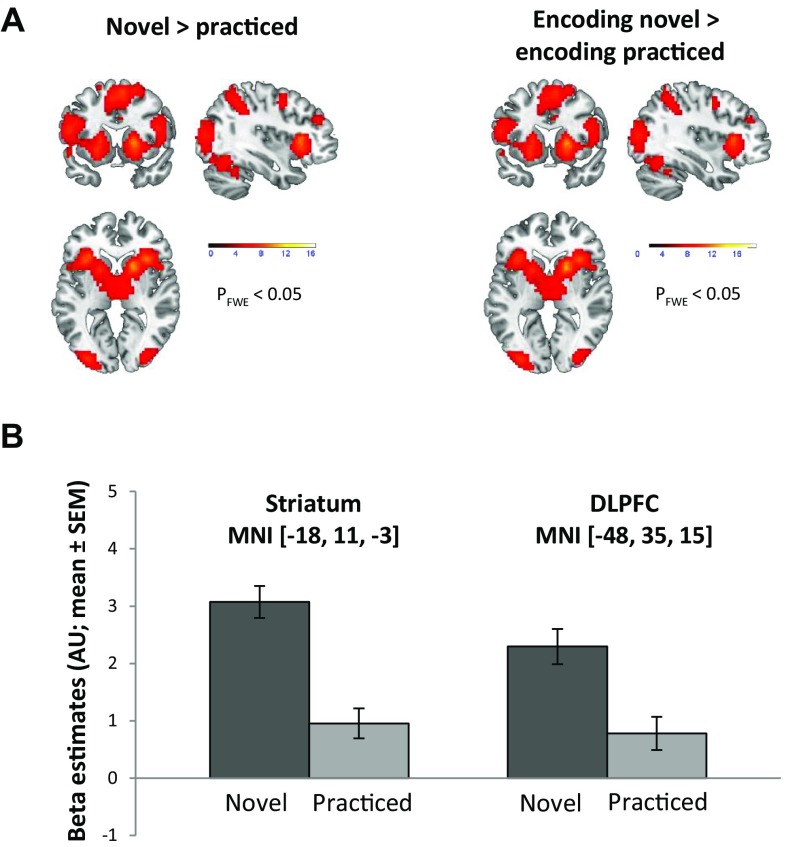
Brain activations at group level. **a** Activation maps for the main effect of stimulus type (novel > practice), and the encoding specific effect of stimulus type (encoding-novel > encoding-practiced). All maps are thresholded at *p* < 0.05, family-wise error corrected (*P*_FWE_) for the whole brain. Color bars represent *t* values. **b** Bar graph illustrations of the mean beta estimates (± SEM) across subjects for the different task conditions within the striatum and DLPFC volumes of interest used for DCM; *AU* arbitrary units

We detected a significant main effect of stimulus type manifesting as a significant bilateral activation increases in the putamen (*t*_max_ = 10.26) and DLPFC (*t*_max_ = 6.1) during the processing of novel relative to practiced stimuli (Fig. 3a). Other significant regions included the anterior insula, anterior cingulate cortex, and higher order motor and visual areas. In the opposite contrast, the comparison of practiced to novel items revealed increased activation bilaterally in the angular gyrus and in the left precuneus (Table 1).

**Table 1:** Whole-brain activations related to stimulus type (*p* < 0.05 family-wise error corrected for the whole brain)

Region (Brodmann area)	Cluster size	*t* value	Peak MNI coordinates		
			*x*	*y*	*z*
Novel > practice					
Insula (BA 13)	4106	10.34	33	23	6
Anterior putamen		10.26	18	14	6
SMA (BA 6)		9.67	6	8	60
Insula (BA 13)		8.82	− 36	23	3
Middle cingulum (BA 32)		8.49	9	20	36
Anterior putamen		8.35	− 18	8	6
SMA (BA 6)		7.99	− 3	11	54
Middle cingulum (BA 32)		7.99	− 6	17	42
Precentral gyrus (BA 6)		7.82	− 42	− 7	48
Precentral gyrus (BA 6)		7.51	48	− 1	45
Thalamus		7.22	6	− 4	3
Thalamus		6.47	− 6	− 7	3
DLPFC (BA 10/46)		6.1	− 45	32	18
Superior parietal gyrus (BA 7)	1399	8.95	27	− 64	45
Parietal inferior gyrus (BA 40)		7.07	45	− 40	48
Middle occipital gyrus (BA 18/19)		7.51	36	− 85	12
Inferior occipital gyrus (BA 20/37)		8.06	45	− 61	− 15
Fusiform gyrus (BA 19/37)		8.06	45	− 61	− 15
Superior parietal gyrus (BA 7)	1560	8.85	− 24	− 64	54
Middle occipital gyrus (BA 18/19)		8.6	− 27	− 76	24
Parietal inferior gyrus (BA 40)		7.55	− 42	− 40	42
Inferior occipital gyrus (BA 20/37)		6.72	− 48	− 61	− 15
Fusiform gyrus (BA 19/37)		7.19	− 30	− 64	− 12
DLPFC (BA 10/46)	39	6.08	39	41	27
Cerebellum	12	5.94	− 39	− 58	− 24
Cerebellum	19	5.72	36	− 52	− 27
Practice > novel					
Precuneus (BA 31)	109	6.68	− 6	− 58	27
Angular gyrus (BA 39/40)	127	6.53	− 51	− 70	39
Angular gyrus (BA 39/40)	31	6.11	54	− 67	36

Regions were classified according to the Automated Anatomical Labeling Atlas (Tzourio-Mazoyer et al. 2002). Coordinates (in Montreal Neuroimaging (MNI) space) and statistical information refer to the peak voxel in the corresponding area. Cluster size is given at *p* < 0.05 (family-wise error corrected for the whole brain)

*SMA* supplementary motor areas, *dPMC* dorsal premotor cortex, *DLPFC* dorsolateral prefrontal cortex

For the encoding specific effect of stimulus type we observed a relative increase of activation during encoding of novel relative to practiced stimuli in the putamen (*t*_max_ = 10.43) and several other cortical and subcortical areas including the DLPFC (Fig. 3a). The opposite contrast revealed activation in the bilateral angular gyrus and left precuneus (Table 2).

**Table 2:** Whole-brain activations related to encoding specific effects of stimuli type (p < 0.05 family-wise error corrected for the whole brain). See Table 1 legend for details on the classification of regions, coordinates, statistics, and abbreviations

Region (Brodmann area)	Cluster size	*t* value	Peak MNI coordinates		
			*x*	*y*	*z*
Novel (encoding > retrieval) > practiced (encoding > retrieval)					
Anterior putamen	3536	9.43	21	14	6
Insula (BA 13)		8.8	33	23	6
Pre-SMA (BA 6)		8.23	6	5	63
dPMC/DLPFC (BA 6/9)		7.51	48	− 1	45
Anterior putamen		7.42	− 18	8	3
Middle cingulum (BA 32)		7.38	9	20	36
Insula		7.29	− 36	23	3
Middle cingulum (BA 32)		6.98	− 6	17	39
Pre-SMA		6.82	− 3	5	63
Inferior frontal gyrus (BA 44/45)		6.72	− 54	8	18
Inferior frontal gyrus		6.41	48	11	18
dPMC/DLPFC (BA 6/9)		6.03	− 51	− 1	36
Thalamus		5.41	9	− 7	0
Thalamus		5.29	− 6	− 10	0
Middle occipital gyrus (BA 18/19)	1228	7.76	36	− 85	12
Inferior occipital gyrus (BA 20/37)		6.86	45	− 64	− 6
Parietal inferior gyrus (BA 40)		5.38	45	− 37	48
Fusiform gyrus (BA 37)		5.15	39	− 61	− 12
Middle occipital gyrus (BA 18/19)	1179	7.57	− 36	− 88	3
Superior parietal gyrus (BA 7)		7.31	− 21	− 64	54
Fusiform gyrus (BA 37)		6.66	− 30	− 64	− 12
Superior parietal gyrus (BA 7)		6.51	27	− 61	51
Inferior occipital gyrus (BA 20/37)		5.62	− 45	− 73	− 6
Parietal inferior gyrus (BA 40)	102	5.34	− 39	− 40	45
Practice (encoding > retrieval) > novel (encoding > retrieval)					
Angular gyrus (BA 39/40)	100	5.42	− 48	− 70	42
Precuneus (BA 31)	51	5.36	− 6	− 58	24
Angular gyrus (BA 39/40)	24	5.22	54	− 64	36

For all contrasts revealing striatal activations, i.e., contrasts reflecting increased activation during encoding and during the processing of novel items, clusters were located in the anterior putamen, i.e., dorsal and rostral to the anterior commissure. For illustration purposes, Fig. 3b depicts the response profile of the DLPFC and putamen within the VOIs that were subsequently used for DCM.

### Dynamic causal modeling

BMS analysis of the model families, which varied by modulation pattern, identified a clear winning family (model family # 16; family exceedance probability: *p* = 0.94). This model family comprised a full modulation pattern, i.e., a modulation of both intrinsic connections (DLPFC to striatum and striatum to DLPFC) by the two encoding conditions (EN, EP; Fig. 2b).

Within the winning family, the highest protected exceedance probability was observed for the model including the input from all four conditions exclusively to DLPFC (model # 9; protected exceedance probability: *p* = 0.86; Fig. 2b, c).

Further inspection of the winning model parameters using BPA revealed a significant input from encoding novel and encoding practice to DLPFC as well as a significant top-down connection from DLPFC to striatum. Further, we found a significant modulatory effect for encoding novel on the top-down connection from DLPFC to striatum (*p* = 1). Bayesian parameter averages including posterior probabilities of the winning model are shown in Table 3. The winning model including significant BPA results is displayed in Fig. 2c. In order to rule out an effect of the thalamus on DLPFC-driven top-down modulation of the striatum (Perakyla et al. 2017), we performed a supplemental DCM analysis including the thalamus as a VOI, which had no impact on the main outcome of our initial analysis (i.e., modulation of the connection from DLPFC to striatum, see supplementary materials for details).

**Table 3:**
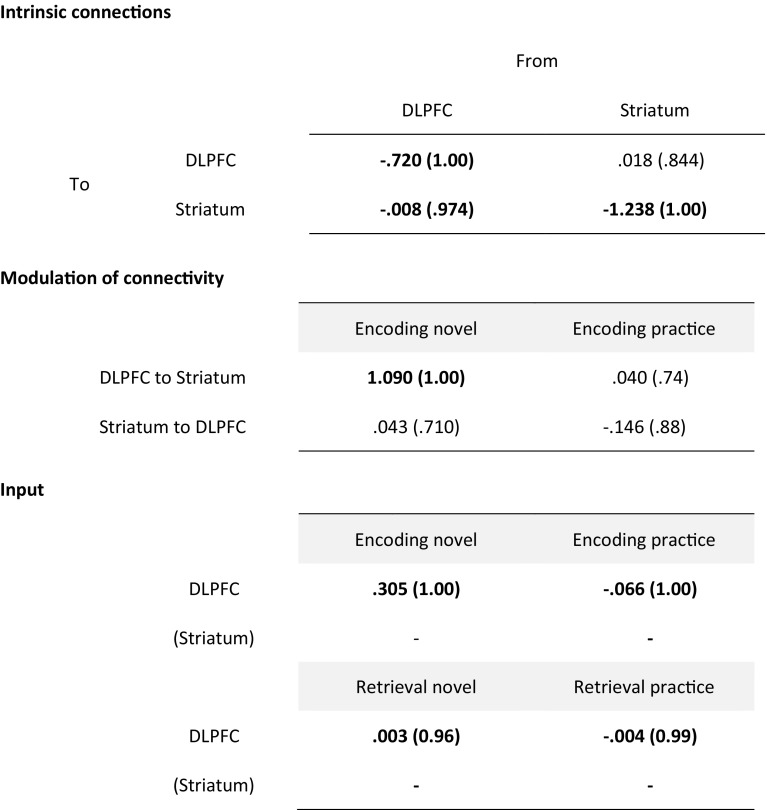
Bayesian parameter averages of the winning model with posterior probabilities in parentheses and significant parameters in bold print

Note that the winning model did not include any input to the striatum

## Discussion

The cognitive loop of the cortical–striatal circuitry is anatomically well-defined (Alexander et al. 1986; Draganski et al. 2008; Lehericy et al. 2004) and has been implicated in the control of the access of relevant materials into working memory (Gruber et al. 2006; McNab and Klingberg 2008; O’Reilly and Frank 2006). While the admission concurs to the encoding stage of the task, a plausible aspect of cognitive relevance is the degree of novelty of the encoded items. However, the current knowledge on the effects of working memory task stage and stimulus novelty on frontal–striatal activation is still limited and a critical role of the encoding phase for the modulation of frontal–striatal effective connectivity during novelty processing has not been established. To address these questions, we used fMRI and DCM in a sample of 74 healthy volunteers performing a modified Sternberg working memory task allowing for the study of different task phases and the degree of stimulus novelty. As our main findings, we report a highly significant engagement of the anterior putamen and DLPFC along with probabilistic evidence for an increase in DLPFC–putamen effective connectivity during the encoding of novel working memory items.

Our data confirm and extend several aspects of prior neuroimaging work in this area. Firstly, in line with prior reports (Jansma et al. 2001; van Raalten et al. 2008a, b), the behavioral response to novel working memory items was significantly slower and less accurate. This observation is consistent with the theory that previously practiced stimuli invoke the execution a well-developed and automatic skill, while novel items demand a more controlled, effortful and capacity-limited mode of information processing (Shiffrin and Schneider 1977).

Secondly, again consistent with prior work, we observed an increase in activation in working memory-related cortical regions such as the left DLPFC, left anterior insula, superior parietal cortex, anterior cingulate cortex, and the pre-supplementary motor area during the processing of novel relative to practiced Sternberg items (Jansma et al. 2001; van Raalten et al. 2008a). Such activation increases have previously been explained by the inability for a capacity-relieving “chunking”—or binding of separate stimulus–response associations in a higher order representation with fewer information elements—in the context of novel and continuously changing materials (Guida et al. 2012; Jansma et al. 2001; Landau et al. 2004). Moreover, as in prior studies with short-term training regimens, the detected activation differences in working memory-related cortical areas were quantitative rather than qualitative in nature. This opinion is supported by the observation that short-term training led to an activation decrease in the very same areas that were engaged during the processing of novel Sternberg items while practice-related activation increases in other regions were not detected (Guida et al. 2012; Landau et al. 2004). A plausible explanation that has been offered for this observation is that short-term acquisition of working memory is restrained to chunk formation within frontal–parietal areas, and that the novelty-induced increase in regional activation reflect a decrease in neural efficiency and cognitive capacity (Guida et al. 2012). It should be noted, however, that while our experiment was set out to assess the effects of novelty in the context of working memory processing, we cannot rule out an additional modulation of corticostriatal responses by long-term memory representations in the context of the trained (as compared to the novel) stimulus sets. Despite similar sensory and motor requirements, the comparison of novel to practiced conditions additionally revealed activation in higher-level motor and visual areas. The involvement of these areas is in line with well-known downstream effects of top-down control mechanisms (e.g., attention) and is reflective of the higher degree of complexity/task difficulty of novel as compared to practiced trials (Gilbert and Li 2013; Hertrich et al. 2016).

Thirdly, our data extend prior knowledge by demonstrating a highly significant engagement of the frontal–striatal circuitry during working memory, which was selective for the encoding relative to the retrieval phase of the task. Here, the effect was mostly explained by an activation increase during the encoding phase of the cognitively more demanding novel stimuli, for which a higher-order representation through “chunking” was prevented by the continuous changes in stimulus–response relationships and the related requirements for frequent updating. Notably, the detected activation foci in the putamen were dorsal and rostral to the anterior commissure, consistent with a recruitment of the associative (i.e., cognitive) territories of the cortical-striatal circuitry (Alexander et al. 1986). Although prior working memory studies have mostly focused on effects within frontal-parietal areas (Guida et al. 2012), activation increases in the dorsal anterior putamen have been related to the encoding of stimuli with a higher cognitive load (Chang et al. 2007; Landau et al. 2004), increased level of abstraction (Nee and Brown 2013), or the requirement for frequent updating (Dahlin et al. 2008). In addition, more ventral (but occasionally also dorsal) responses in the anterior striatum have been associated with the presentation of novel or surprising non-rewarding stimuli (Murty et al. 2013; van Schouwenburg et al. 2010; Wittmann et al. 2008; Zink et al. 2003) and related shifts in attention (van Schouwenburg et al. 2010).

Taken together our data support a role for the anterior striatum in the stimulus encoding process that goes beyond the traditional roles of the basal ganglia in motor control and reward processing. Computational models propose that the mechanisms of the basal ganglia, by which it supports working memory, are evolved implementations of the same basic machinery supporting the gating of adaptive responses in the “more primitive” striatal motor circuitry (O’Reilly and Frank 2006). Indeed, the increased engagement of the anterior putamen during the encoding of novel Sternberg materials may serve as a critical gating signal for prefrontal working memory buffers. It may indicate, for example, that the presented items are behaviorally relevant and require a more controlled and resourceful neural strategy for their effective handling. Conversely, the relative absence of striatal responses during the encoding of practiced stimuli may signal the availability of “pre-chunked rule sets” or automatized neural representations in the frontal-parietal cortex which allow for their efficient neural processing.

Fourthly, the complemental DCM analysis extends our activation findings by highlighting a potential mechanism by which the proposed corticostriatal gating function during the processing of novel working memory items might be achieved. Here, our data were best explained by a model assuming a significant intrinsic connectivity from DLPFC to anterior striatum, as well as an enhancing significant modulatory effect of the encoding phase of novel items on the connection from the DLPFC to the striatum. In general, this observation is in good agreement with the known excitatory projections from the cortex to the striatum (Alexander et al. 1986), and the role of the DLPFC in the top-down control of subcortical structures. We propose that this modulation of prefrontal–striatal connectivity reflects a mechanism by which the DLPFC signals the need for a more resourceful neural strategy (or absence of “pre-chunked cortical rule sets”) for the successful handling of novel items during stimulus encoding. Although such a strategy could plausibly facilitate the enhanced signaling (or gating of relevant stimulus information) in the anterior striatum, further research is needed to substantiate this proposal. Of note, we observed a negative driving input of the practice conditions to the DLPFC, an observation that parallels the relative reduction of the activation of the region in these conditions. Similar to prior reports (Jung et al. 2018) we interpret these findings as related, i.e., the reduction of the overall activity in DLPFC during the practice condition as a consequence of the negative driving input, which may be the result of an interactions between excitatory and inhibitory interneurons in the region.

In summary, we combined a well-established Sternberg working memory task with fMRI activation analyses and DCM to demonstrate a highly significant engagement of the anterior striatum and selective positive modulation of the connection from the DLPFC to the anterior striatum in the context of the encoding of novel working memory materials. Our data further underscore the relevance of the anterior striatum for human cognitive function, further support a role of the basal ganglia as a functional gate for relevant information across behavioral domains, and provide a mechanistic explanation on how the DLPFC may facilitate the input-gating of novel working memory materials through top-down control of the downstream basal ganglia.

## Electronic supplementary material

Below is the link to the electronic supplementary material.

Supplementary material 1 (PDF 368 KB)
