# Supplementary material for: Novelty modulates human striatal activation and prefrontal–striatal effective connectivity during working memory encoding
Source: Brain Struct Funct. 2018 May 11;223(7):3121–32. doi: 10.1007/s00429-018-1679-0 (PMC6132644; doi:10.1007/s00429-018-1679-0)
Supplement: Supplementary file 1 — Supplementary material 1 (PDF 368 KB) [file 429_2018_1679_MOESM1_ESM.pdf]

Supplementary material - Brain Structure and Function

Novelty modulates human striatal activation and prefrontal-striatal effective connectivity during working memory encoding

Lena S. Geiger[1], Carolin Moessnang[1], Axel Schäfer[1], Zhenxiang Zang[1], Maria Zangl[1], Hengyi Cao[1], Tamar R. van Raalten[2], Andreas Meyer-Lindenberg[1], Heike Tost[1]

[1]Central Institute of Mental Health, Medical Faculty Mannheim, University of Heidelberg, Mannheim, Germany
[2]Department of Psychiatry, Rudolf Magnus Brain Center, University Medical Center Utrecht, Utrecht, The Netherlands

Corresponding author: Heike Tost, M.D. Ph. D.; E-Mail: heike.tost@zi-mannheim.de

Supplemental results – main effect of working memory phase

For the main effect of working memory phase, we detected a significant bilateral activation increase in the putamen (t_{max} = 10.98) and DLPFC (t_{max} = 9.99) during the encoding relative to the retrieval of working memory items (Figure S1). Other regions surviving whole-brain correction (p_{FWE} < 0.05) included the anterior insula, middle temporal gyrus, anterior cingulate cortex, hippocampus, and higher order motor and visual areas (Table S1). In the opposite contrast, a single cluster in the left postcentral gyrus was observed (Table S1).

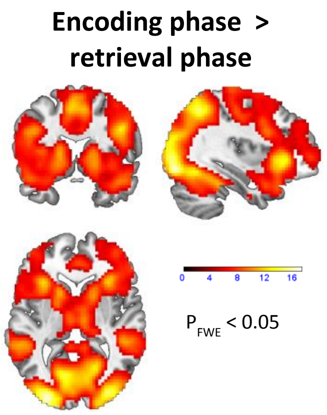

Figure S1: Activation maps for the main effect of working memory phase (encoding > retrieval), thresholded at *p* < 0.05, family-wise error corrected (FWE) for the whole brain. Color bar represents t values. Please note that the interpretation of this contrast is limited by the differences in visual stimulation, task demands, and duration between both task phases.

TABLE S1. Regional brain activation related to the main effect of memory phase

Region (Brodmann area)	Cluster size	t value	Peak MNI coordinates		
			x	y	z
Encoding > retrieval					
Middle occipital gyrus (BA 18)	30.559	17.04	-30	-94	9
Inferior occipital gyrus (BA 19)		16.39	33	-85	-9
Inferior occipital gyrus (BA 19)		16.14	-39	-82	-9
Fusiform gyrus (BA 19/37)		15.84	42	-64	-12
Fusiform gyrus (BA 19/37)		15.68	-39	-70	-12
Middle Cingulum (BA 23)		15.42	-3	-31	33
Precuneus (BA 7)		15.38	27	-64	45
Middle occipital gyrus (BA 18)		14.51	30	-88	-12
Middle Cingulum (BA 23)		14.16	3	-31	33
Precuneus (BA 7)		13.98	-27	-61	51
Insula (BA 13)		13.72	-33	23	0
Precentral gyrus (BA 4)		13.36	-48	-7	42
Insula (BA 13)		13.22	36	26	-3
Middle temporal gyrus (BA 22)		13.1	-54	-34	0
Anterior Cingulum (BA 32)		13.1	12	26	27
Superior temporal gyrus (BA 39/40)		13.1	-54	-34	0
Middle temporal gyrus (BA 22)		12.98	48	-28	-3
Superior temporal gyrus (BA 39/40)		12.98	48	-28	-3
SMA (BA 6)		12.71	-3	5	63
Hippocampus		12.35	-21	-31	-6
Anterior Cingulum (BA 24/32)		12.34	-9	32	24
Precentral Gyrus (BA 4)		11.67	51	-1	42
Hippocampus		11.2	21	-31	-6
Thalamus		11.01	6	-10	0
Anterior Putamen		10.98	-18	14	3
SMA (BA 6)		10.81	3	5	63
Anterior Putamen		10.64	18	14	0
Thalamus		10.35	-6	-13	0
DLPFC (BA 10/46)		9.99	51	35	15
Parietal inferior gyrus (40)		9.81	42	-58	48
Cerebellum/Vermis		9.11	-3	-73	-27
DLPFC (10/46)		8.27	-48	35	15
Cerebellum		7.92	-27	-64	-30
Parietal inferior gyrus (BA 40)		7.55	-42	-43	45
Inferior frontal gyrus (BA 44/45)		7.05	-54	17	6
Cerebellum		6.52	33	-61	-33
Inferior frontal gyrus (BA 44/45)		6.22	51	23	3
Retrieval > novel					
Postcentral gyrus (BA3)	24	5.69	-39	-28	57

Table S1 Whole-brain activations related to memory phase ($p < 0.05$ family-wise error corrected for the whole brain). Regions were classified according to the Automated Anatomical Labeling Atlas (Tzourio-Mazoyer et al., 2002). Coordinates (in Montreal Neuroimaging (MNI) space) and statistical information refer to the peak voxel

in the corresponding area. Abbreviations: supplementary motor areas (SMA), dorsal premotor cortex (dPMC), dorsolateral prefrontal cortex (DLPFC)

Supplementary Methods – Distribution of individual VOIs

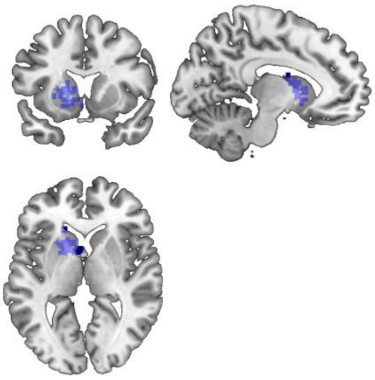

Figure S2: Distribution of individual peak voxels within the anatomical striatum mask (ROI mask) superimposed on T1-weighted images. For DCM VOI definition, 6-mm spheres were constructed around individual peaks.

Supplemental DCM analysis

In order to rule out that our DCM finding was dependent on an unaddressed influence of the thalamus, we performed a supplemental DCM analysis including a VOI for the thalamus as suggested by the reviewer. The subject-specific thalamic VOIs were defined as follows: First, in order to ensure regional specificity, we derived a mask from the group peak activation of the “encoding novel > encoding practiced” contrast by centering a 6-mm sphere around the peak voxel within a structural ROI of the thalamus (AAL atlas). This empirically defined thalamus mask was used to subsequently localize the individual peak voxel in each subject’s contrast image and to extract the first eigenvariate from 6-mm spheres centered around this individual peak.

Second, the thalamus was included as additional region using the same connection and modulation scheme as in the winning model described in the manuscript. We then determined the location of the input by varying the input to the striatum, DLPFC, and thalamus, respectively, which resulted in three full models. Random effects Bayesian model selection (BMS) analysis yielded a winning model with highest protected exceedance probability for the input to the DLPFC ($p = .83$), followed by the striatum ($p = .122$), and the thalamus ($p = .05$). Subsequent Bayesian parameter averaging (BPA) suggests that the modulation of the connection from DLPFC to striatum is robust to the inclusion of the thalamus in the model (see table S2, all parameters shown have evidence > .95).

TABLE S2. Bayesian parameter averages of the winning model

**Intrinsic connections**		From		
		DLPFC	Striatum	Thalamus
To	DLPFC	-.74	-.03	-.02
	Striatum	-.04	-1.30	-.14
	Thalamus	.03	-.03	-1.54

Modulation of connectivity

		Encoding novel		
		From		
		DLPFC	Striatum	Thalamus
To	DLPFC	-	0.87	-1.28
	Striatum	1.33	-	.64
	Thalamus	.29	.47	-

		Encoding practiced		
		From		
		DLPFC	Striatum	Thalamus
To	DLPFC	-	n.s.	-.85
	Striatum	.14	-	1.46
	Thalamus	n.s.	.49	-

Input

		Encoding novel	Encoding practiced
To	DLPFC	.24	-.09
